# Key *Ochsner Journal* Metrics Increased Year Over Year

**DOI:** 10.31486/toj.25.5053

**Published:** 2025

**Authors:** Ronald G. Amedee

**Affiliations:** Director of Clinical School and Professor, The University of Queensland Medical School, Ochsner Clinical School, New Orleans, LA; Editor-in-Chief, *Ochsner Journal*

*Spring won’t let me stay in this house any longer! I must get out and breathe the air deeply again.*

*–Gustav Mahler*

The Spring 2025 issue of *Ochsner Journal* contains four original research articles and five case reports and clinical observations. In addition, the issue contains an innovative program submission entitled “Partnering With Schools for Community-Based Health Interventions: How Educating Children Improves Hypertension Awareness” authored by Hundley, Olson, Rocha, Wallace, Smith, Martin, Crane, D’Agostino, Ladd, and Shah. Dr Sangeeta Shah is a former Ochsner Health cardiologist now practicing at Virginia Commonwealth University Medical Center. This paper is followed by a quality improvement article authored by Ochsner Health physicians Sumrall and Gilly, as well as Jakob Oury, a 4th-year University of Queensland-Ochsner Clinical School (UQ-OCS) medical student, entitled “Enhancing Physician Satisfaction and Patient Safety Through an Artificial Intelligence–Driven Scheduling System in Anesthesiology.”

Our first original research article was written by Newcomb, Urvater, Doig, Mullen, and Cooke and reviews the “Effect of Weekend Admission on Hip Fracture Mortality.” The first author of this paper, Nicholas Newcomb, is a former UQ-OCS medical student and is now completing his orthopedics residency at the University of New Mexico, Albuquerque. Marlena Urvater is a current UQ-OCS student. The second original research article provides a “Comparative Analysis of Ultrasound-Guided Pain Management Approaches for Sternotomy in Cardiac Surgeries—Transversus Thoracic Muscle Plane Block vs Pecto-Intercostal Fascial Block” and is authored by Vanjare, Deshmukh, Barasker, Kassim, and Arya. “Evaluating the Role of the Jaw Thrust Maneuver During Tracheal Intubation in Reducing the Incidence of Postoperative Sore Throat: A Prospective Randomized Study” was submitted by Saxena, Rathore, P Jain, A Jain, and Barasker. Completing the original research section is an article by Ochsner authors Ashcraft, St Pierre, Davis, Scariano, Latsis, Vortisch, Smith, and Pankey on “Evaluation of the Diagnostic Accuracy of the T2Resistance Panel (Research Use Only) in Patients With Possible Bacterial Bloodstream Infections.”

The case reports and clinical observations section begins with “Cerebellar Ataxia With Neuropathy and Bilateral Vestibular Areflexia Syndrome Coexisting With JAK2-Positive Polycythemia Vera and Myelofibrosis” by Saibaba, Selvaraj, Viswanathan, and Pillai. Next is “Following Multiple Failed Reconstructions of a Distal Femur Fracture, Osseous Union Achieved After Superficial Femoral Artery Endarterectomy” by Johns, Curtis, and Gehlert. “Management of Spontaneous Renal Arteriovenous Fistula in Pregnancy” was authored by an Ochsner Health team of Tamakloe (former UQ-OCS student), Davey, Dorn, Gilbert, and Williams. This case is followed by the report “Excessive Ingestion of Almond Milk Causes Severe Hypercalcemia and Acute Kidney Injury in a Patient With Chronic Kidney Disease” written by a team that includes two Ochsner Health nephrologists, Mohamed and Velez. The final case report from Malik, Goyal, Gupta, and Bhagat presents a patient with “Idiopathic Arginine Vasopressin Deficiency With Mild and Reversible Hypercalcemia.”

This issue of *Ochsner Journal* is published in March 2025 as springtime arrives in New Orleans. Our recent winter chill included 10 to 12 inches of snowfall which was a most unusual sight for Southerners to behold. Spring is welcome!

*Ochsner Journal* is a quite unique publishing model. The *Journal* is fully funded by Ochsner Health, charges no fees to authors, and is completely open access with no paywall, ensuring that the full text of all articles is available for everyone to read. As the breadth of topics in the Spring issue makes evident, *Ochsner Journal* is a general medicine publication that aims to accommodate submissions from all specialties. Each published article is assigned a digital object identifier (DOI) and a PubMed ID. All *Ochsner Journal* articles are discoverable at PubMed, Web of Science, Google Scholar, Semantic Scholar, and other databases.

Year-over-year metrics (January 2024 vs January 2025) for *Ochsner Journal* show increases in four key areas. The *Journal*’s h-index rose from 71 to 76, and the total number of external citations increased from 26,623 to 31,494 (data from Google Scholar). Our Scopus CiteScore increased from 2.0 to 2.1 (data from Elsevier), and finally, the *Journal*’s impact factor rose from 1.2 to 1.3 (data from Clarivate). These metrics are all signs that *Ochsner Journal* is a successful, visible, and viable scholarly product that continues to enhance the reputation of Ochsner Health. Thank you in advance for all submissions. Please keep them coming as quality papers are the lifeblood of any publication.

